# Changes in vaping trends since the announcement of an impending ban on disposable vapes: A population study in Great Britain

**DOI:** 10.1111/add.70057

**Published:** 2025-04-15

**Authors:** Sarah E. Jackson, Lion Shahab, Harry Tattan‐Birch, Vera Buss, Jamie Brown

**Affiliations:** ^1^ Department of Behavioural Science and Health University College London London UK; ^2^ SPECTRUM Consortium Edinburgh UK

**Keywords:** disposable vape ban, disposable vapes, e‐cigarettes, population trends, tobacco and vapes bill, vaping

## Abstract

**Background/Aim:**

There has been a rapid rise in vaping prevalence among youth and young adults in Great Britain since disposable vapes started to become popular in 2021. In January 2024, the government announced plans to introduce new vaping policies, including a ban on disposable vapes, to tackle youth vaping. This study measured whether trends in current vaping and use of disposable vapes have changed since this announcement.

**Methods:**

Segmented regression analysis of data collected monthly between January 2022 and January 2025 as part of the Smoking Toolkit Study, a representative household survey in Great Britain. We ran generalised additive models using data from all participants aged ≥16y (*n* = 88 611; ‘adults’) and from a subset aged 16‐24y (*n* = 9276; ‘young adults’). Main outcome measures were changes in trends in (a) the prevalence of current vaping and (b) the proportion of vapers mainly using disposable devices.

**Results:**

Before January 2024, vaping prevalence was increasing by 23.4% per year [relative risk (RR)_trend_ = 1.234, 95% confidence interval (CI) = 1.184–1.287] and use of disposable vapes was increasing by 17.7% per year (RR_trend_ = 1.177, 95% CI = 1.105–1.255). These trends changed after the new policy measures were announced (RR_Δtrend_ = 0.782, 95% CI = 0.696–0.878, and RR_Δtrend_ = 0.573, 95% CI = 0.487–0.673, respectively). Instead of increasing, vaping prevalence stabilised and there was a substantial decline in the proportion of vapers mainly using disposables from 43.6% (40.1–47.3%) in January 2024 to 29.4% (26.3–32.9%) in January 2025. Similar changes were observed among young adults (vaping prevalence: RR_Δtrend_ = 0.799, 95% CI = 0.645–0.990; use of disposable vapes: RR_Δtrend_ = 0.547, 95% CI = 0.435–0.688), with vaping prevalence stabilising between January 2024 and January 2025 and the proportion of vapers mainly using disposables falling from 63.2% (58.8–67.8%) to 35.2% (30.4–40.8%).

**Conclusions:**

Following the announcement of an impending ban on disposable vapes and other potential vaping policies, recent increases in vaping prevalence in Great Britain stalled, including among young adults. In addition, there was a shift away from using disposable vapes, with people increasingly opting to use devices that can be refilled and recharged.

## INTRODUCTION

The prevalence of e‐cigarette use (‘vaping’) among youth and young adults in Great Britain has risen rapidly since 2021 [1, 2, 3, 4]. This increase appears to have been driven by the introduction of new disposable vapes, which are discarded after the battery or e‐liquid runs out [1, 2, 3, 5, 6]. These products are cheap and easy to use, have sleek design and colourful branding and are widely available from a range of retail outlets [7, 8, 9]. As of 2023, most young vapers were using disposables [3, 4], as were approximately half of adults who had been vaping for 6 months or more [4]. Concerns over their appeal to youth and their environmental impact [10] led to calls for an outright ban on disposable vapes [11, 12, 13].

In October 2023, the United Kingdom (UK) Government published a command paper outlining a range of measures it was considering to reduce youth vaping, including restricting the sale of disposable vapes [14]. Following a 6‐week consultation, the Prime Minister announced in January 2024 that disposable vapes would be banned in the United Kingdom as part of a package of measures ‘to tackle the rise in youth vaping and protect children's health’ [15]. In addition to reducing youth vaping, another important rationale for banning disposables was that it would have benefits for the environment [15]. This announcement attracted widespread media coverage [16].

New legislation to ban the sale of disposable vapes in England was developed under the Environmental Protection Act 1990 and will come into effect from 1 June 2025 [17, 18]. The Scottish and Welsh Governments have also confirmed that they will align with England and introduce a ban on disposables on the same date [19, 20]. Other measures to tackle youth vaping are currently progressing through parliament as part of the Tobacco and Vapes Bill [21]. These include powers to restrict the way e‐cigarettes are formulated (e.g. the flavours they can contain), branded (e.g. the way they are packaged), marketed and sold (e.g. the way they are displayed in shops), all of which will be subject to consultation and secondary legislation.

However, the vaping landscape evolves rapidly and changes in behaviour are often seen before legislation comes into effect [22]. E‐cigarette manufacturers responded to news of the ban by starting to sell ‘disposable‐like’ products: reusable versions of their popular disposable models. Notifications to the Medicines and Healthcare products Regulatory Agency for disposable products quickly declined following the announcement of the ban, while notifications for products with similar branding and features to those in disposable category but that were newly rechargeable or refillable increased [23]. This may have prompted some vapers to transition away from disposable devices. Other vapers may have sought out reusable options after hearing about the impending ban. News coverage of the adverse impacts of disposable vapes on the environment may have increased the salience of these concerns for some users.

It is important to have up‐to‐date information to inform policy decisions. This study aimed to establish whether there have been changes in e‐cigarette use in the months since the government announced its intention to introduce new vaping policies. Specifically, we aimed to examine changes in monthly trends in (a) vaping prevalence and (b) the proportion of vapers who report mainly using disposable devices, among people in Great Britain age 16 years or older (‘adults’) and specifically among those age 16 to 24 years (‘young adults’). We also explored changes in these trends among adults stratified by smoking status.

## METHODS

### Design

Data were drawn from the Smoking Toolkit Study, an ongoing monthly cross‐sectional survey of a representative sample of adults (≥16 years) in Great Britain [24, 25]. The study uses a hybrid of random probability and quota sampling to select a new sample of approximately 2450 adults each month. Data are collected through telephone interviews. Comparisons with other national surveys and sales data indicate the survey achieves nationally representative estimates of key socio‐demographic and nicotine use variables [24, 26].

The present analyses used data from respondents in the period from January 2022 (~6 months after disposable vapes started to become popular [1] and 2 years before the government's announcement of new potential vaping policies [15]) to January 2025 (the most recent data available at the time of analysis; updated from November 2024 following peer review with a similar pattern of results). Data on the main device type used were not collected in England in May, June or August 2022, and these waves were, therefore, excluded from analyses of trends in the use of disposable vapes.

### Measures

Vaping status was assessed within several questions asking about use of a range of nicotine products. Participants who reported current smoking were asked ‘Do you regularly use any of the following in situations when you are not allowed to smoke?’ and those who reported cutting down ‘Which, if any, of the following are you currently using to help you cut down the amount you smoke?’; those who currently smoked or who had quit in the past year were asked ‘Can I check, are you using any of the following either to help you stop smoking, to help you cut down or for any other reason at all?’; and non‐smokers were asked ‘Can I check, are you using any of the following?’. Those who reported using an e‐cigarette in response to any of these questions were considered current vapers, else they were considered non‐vapers.

Main device type used was assessed by asking vapers: ‘Which of the following do you mainly use…?’ (a) a disposable e‐cigarette or vaping device (non‐rechargeable); (b) an e‐cigarette or vaping device that uses replaceable pre‐filled cartridges (rechargeable); (c) an e‐cigarette or vaping device with a tank that you refill with liquids (rechargeable); or (d) a modular system that you refill with liquids (you use your own combination of separate devices: batteries, atomizers, etc.). We categorised device types as disposable (response ‘a’) or reusable (responses ‘b–d’). Participants could also respond that they did not know, and these cases were treated as missing.

Smoking status was assessed by asking participants which of the following best applied to them: (a) I smoke cigarettes (including hand‐rolled) every day; (b) I smoke cigarettes (including hand‐rolled), but not every day; (c) I do not smoke cigarettes at all, but I do smoke tobacco of some kind (e.g. pipe, cigar or shisha); (d) I have stopped smoking completely in the last year; (e) I stopped smoking completely more than a year ago; (f) I have never been a smoker (i.e. smoked for a year or more). Responses ‘a–c’ were considered current smoking, ‘d–e’ former smoking and ‘f’ never regular smoking.

### Statistical analysis

These analyses were not pre‐registered and should be considered exploratory. Data were analysed in R v.4.2.2. The Smoking Toolkit Study uses raking (iterative proportional fitting) [27] to create survey weights that match the sample to the population in Great Britain [25]. The following analyses were done on weighted data. Missing cases were excluded on a per‐analysis basis.

We used segmented regression to assess changes in monthly trends in current vaping and mainly using disposable vapes in Great Britain following the government's announcement of new policy measures to reduce youth vaping. We used log‐binomial generalised additive models (GAMs, using the *mgcv* package in R) to model trends before the announcement (underlying secular trend; coded January 2022 = 1 through January 2025 = 37) and the change in the trend (slope) after the announcement (coded 0 up to January 2024 and 1…*n* from February 2024 onward, where *n* was the number of waves after the announcement). A step‐level change was not included as this was deemed implausible. To adjust for seasonality (month‐of‐year effects), we also included a variable reflecting the calendar month coded from January = 1 to December = 12. This variable was modelled using a smoothing term with cyclic cubic splines specified. We assumed a linear trend in log‐prevalence before the announcement (i.e. the proportional change in prevalence month‐on‐month would be stable from January 2022 to January 2024). Because of the relatively short length of the time‐series, we expected negligible differences between log‐linear and linear trends. To explore changes among young adults specifically, we repeated the analyses restricting the sample to participants age 16 to 24 years. We also repeated the analyses among adults stratified by smoking status.

For ease of interpretation, we multiplied model coefficients by 12 to convert results from monthly to annual trends. The post‐announcement trend was derived by summing the pre‐announcement trend and the slope (change in trend after the announcement) on the log scale and exponentiating the result to convert it to a risk ratio (RR). The 95% CI for the post‐announcement trend was derived by combining the uncertainties of both components, calculating the standard error and applying the usual CI formula before exponentiating. We used predicted estimates (using the *predict* function) to plot modelled trends in each outcome alongside unmodelled data points and to estimate the proportion of vapers likely to be mainly using disposables by the time the ban on disposable vapes is implemented in June 2025 (calculated by using the model to predict estimates over an extended time period).

## RESULTS

A total of 88 611 participants were surveyed between January 2022 and January 2025 [mean (SD) age = 48.1 (18.8); 50.6% women], of whom 9276 were 16‐ to 24‐years old [mean (SD) age = 20.7 (2.4); 47.7% women]. There were 7910 vapers surveyed in waves in which the main device type used was assessed, and 4.8% (380/7910) did not know the main device type they used and were excluded from analyses of trends in use of disposable vapes.

Following the announcement in January 2024 of an impending ban on disposable vapes and other potential vaping restrictions, there were notable changes in trends in e‐cigarette use among adults (Table 1) and young adults (Table 2) in Great Britain.

**TABLE 1:** Changes in annual trends in e‐cigarette use among adults (≥16 years) in Great Britain since the announcement of an impending ban on disposable vapes in January 2024.

	Current vaping^a^	Mainly uses disposable vapes^b^
Annual trends, RR [95% CI]		
Pre‐announcement trend (Jan 22–Jan 24)	1.234 [1.184–1.287]	1.177 [1.105–1.255]
Change in annual trend (Jan 24)	0.782 [0.696–0.878]	0.573 [0.487–0.673]
Post‐announcement trend (Jan 24–Jan 25)	0.965 [0.853–1.091]	0.674 [0.567–0.802]
Predicted prevalence estimates, % [95% CI]		
2 years pre‐announcement (Jan 22)	8.9 [8.2–9.6]	31.4 [27.9–35.4]
1 year pre‐announcement (Jan 23)	11.0 [10.3–11.7]	37.0 [34.2–40.1]
Month ban was announced (Jan 24)	13.5 [12.6–14.5]	43.6 [40.1–47.3]
1 year post‐announcement (Jan 25)	13.0 [11.9–14.2]	29.4 [26.3–32.9]
Planned implementation of ban (June 25)	–	24.9 [21.4–29.1]

Abbreviations: Jan, January; RR, risk ratio.

Results shown are derived from segmented regression analyses (using generalised additive models) of data collected from January 2022 to January 2025. Models are adjusted for seasonality.

^a^ Among all respondents (unweighted *n*: 88611 adults).

^b^ Among respondents who reported current vaping in waves that assessed the main device type used (unweighted *n*: 7530 adults).

**TABLE 2:** Changes in annual trends in e‐cigarette use among young adults (16–24 years) in Great Britain since the announcement of an impending ban on disposable vapes in January 2024.

	Current vaping^a^	Mainly uses disposable vapes^b^
Annual trends, RR [95% CI]		
Pre‐announcement trend (Jan 22–Jan 24)	1.249 [1.157–1.349]	1.018 [0.942–1.100]
Change in annual trend (Jan 24)	0.799 [0.645–0.990]	0.547 [0.435–0.688]
Post‐announcement trend (Jan 24–Jan 25)	0.998 [0.795–1.253]	0.557 [0.437–0.710]
Predicted prevalence estimates, % [95% CI]		
2 years pre‐announcement (Jan 22)	17.0 [14.8–19.5]	61.0 [54.7–68.0]
1 year pre‐announcement (Jan 23)	21.2 [19.1–23.6]	62.0 [59.1–65.2]
Month ban was announced (Jan 24)	26.5 [23.5–29.9]	63.2 [58.8–67.8]
1 year post‐announcement (Jan 25)	26.5 [22.8–30.8]	35.2 [30.4–40.8]
Planned implementation of ban (June 25)	–	27.6 [22.2–34.3]

Abbreviation: Jan, January; RR, risk ratio.

Results shown are derived from segmented regression analyses (using generalised additive models) of data collected from January 2022 to January 2025. Models are adjusted for seasonality.

^a^ Among all respondents (unweighted *n*: 9276 young adults).

^b^ Among respondents who reported current vaping in waves that assessed the main device type used (unweighted *n*: 1717 young adults).

**FIGURE 1:**
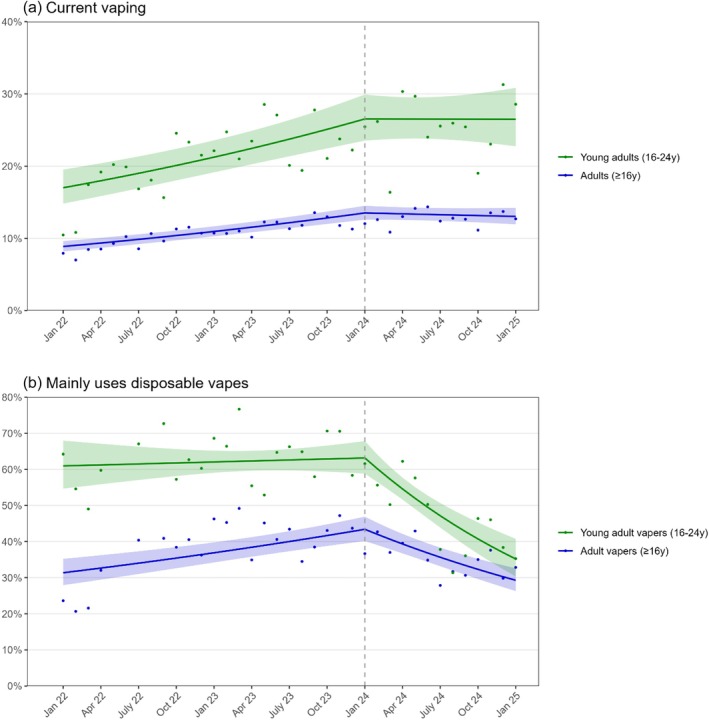
Trends in current vaping and use of disposable vapes, January 2022 to January 2025. Panels show trends in the prevalence of (a) current vaping among adults and young adults, and (b) predominant use of disposable vapes among adult and young adult vapers. The vertical dashed line indicates the timing of the announcement in January 2024 of an impending ban on disposable vapes and other potential vaping restrictions. Points represent unmodelled weighted prevalence by month. Lines represent modelled weighted prevalence over the study period, adjusting for seasonality. Shaded bands represent 95% CI.

Between January 2022 and January 2024, the prevalence of current vaping increased by 23.4% per year among adults from 8.9% in January 2022 to 13.5% in January 2024 and by 24.9% per year among young adults from 17.0% to 26.5% Figure 1(a)]. The proportion of vapers who mainly used disposable devices increased by 17.7% per year among adult vapers from 31.4% to 43.6% and was approximately stable among young adult vapers at a majority of 62.1% (58.2–66.2%) on average [Figure 1(b)].

After the new policy measures were announced in January 2024, trends in current vaping prevalence changed [Figure 1(a)]. Among both adults and young adults, prevalence was no longer increasing and instead remained relatively stable. There was also a substantial downward change in the trends in the use of disposable vapes [Figure 1(b)]. The proportion of vapers mainly using disposable devices declined by 32.6% per year among adult vapers (from 43.6% in January 2024 to 29.4% in January 2025) and by 44.3% per year among young adult vapers (from 63.2% to 35.2%). If current trends continue at the same rate, we project that an estimated 24.9% of adult vapers and 27.6% of young adult vapers would be mainly using disposable vapes by the time the ban on disposables is implemented in June 2025.

**FIGURE 2:**
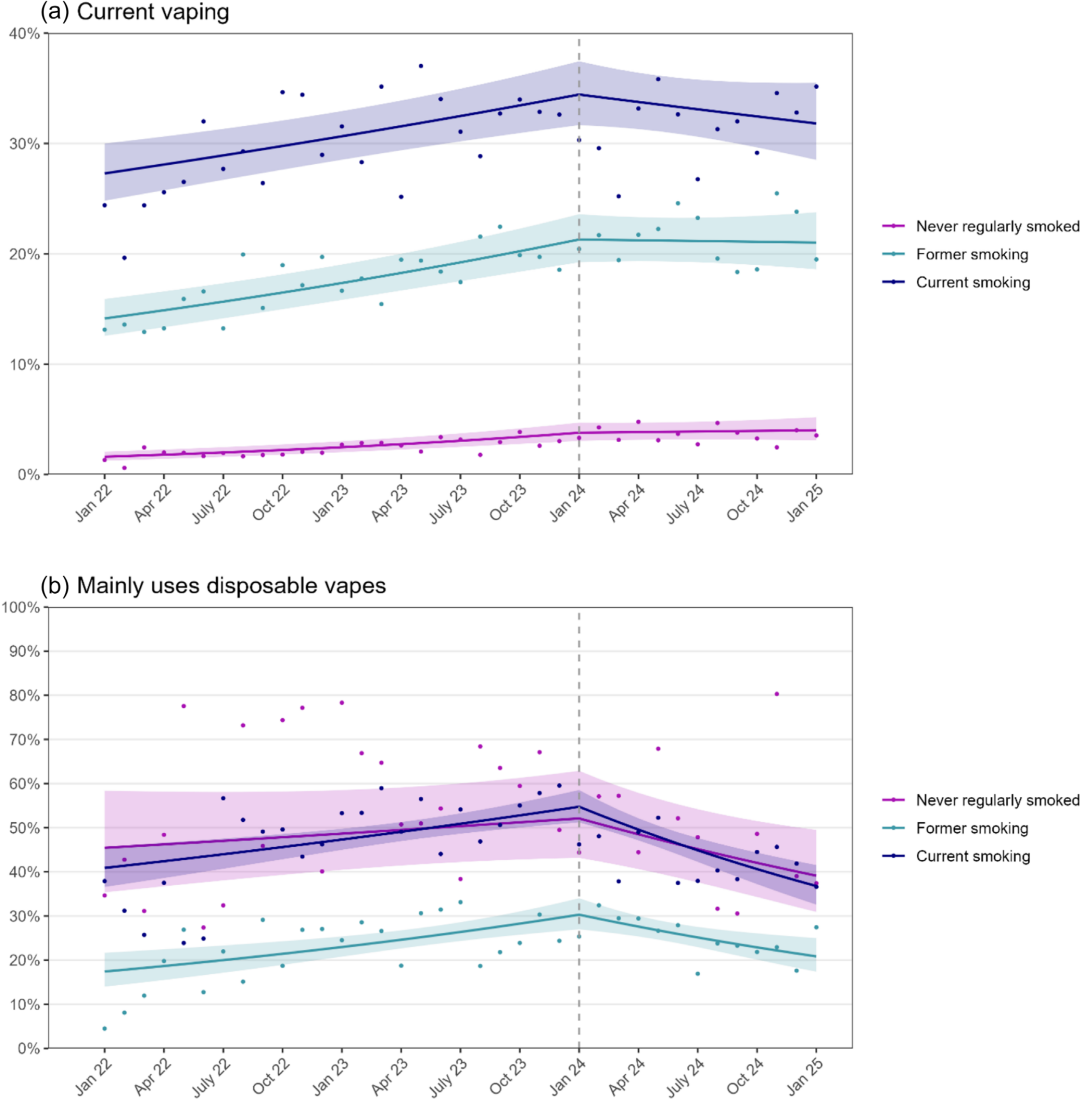
Trends in current vaping and use of disposable vapes by smoking status, January 2022 to January 2025. Panels show trends in the prevalence of (a) current vaping among adults and (b) predominant use of disposable vapes among adult vapers, stratified by smoking status. The vertical dashed line indicates the timing of the announcement in January 2024 of an impending ban on disposable vapes and other potential vaping restrictions. Points represent unmodelled weighted prevalence by month. Lines represent modelled weighted prevalence over the study period, adjusting for seasonality. Shaded bands represent 95% CI.

Trends were similar among adults who reported current smoking, former smoking or having never regularly smoked (Table 3, Figure 2).

**TABLE 3:** Changes in annual trends in e‐cigarette use by smoking status among adults (≥16 years) in Great Britain since the announcement of an impending ban on disposable vapes in January 2024.

Smoking status	Current vaping^a^			Mainly uses disposable vapes^b^		
	Never	Former	Current	Never	Former smokers	Current smokers
Annual trends, RR [95% CI]						
Pre‐announcement trend (Jan 22–Jan 24)	1.532 [1.354–1.732]	1.227 [1.155–1.303]	1.123 [1.065–1.185]	1.071 [0.947–1.211]	1.319 [1.143–1.522]	1.157 [1.072–1.249]
Change in annual trend (Jan 24)	0.691 [0.497–0.961]	0.804 [0.684–0.946]	0.822 [0.706–0.958]	0.701 [0.520–0.946]	0.522 [0.371–0.733]	0.581 [0.473–0.712]
Post‐announcement trend (Jan 24 – Jan 25)	1.059 [0.745–1.505]	0.987 [0.830–1.173]	0.924 [0.786–1.086]	0.751 [0.544–1.037]	0.688 [0.476–0.995]	0.672 [0.540–0.836]
Predicted prevalence estimates, % [95% CI]						
2 years pre‐announcement (Jan 22)	1.6 [1.3–2.1]	14.1 [12.6–15.9]	27.3 [24.8–30.0]	45.4 [35.4–58.4]	17.4 [14.0–21.6]	40.9 [36.6–45.7]
1 year pre‐announcement (Jan 23)	2.5 [2.0–3.0]	17.4 [15.8–19.0]	30.7 [28.5–32.9]	48.7 [40.5–58.5]	23.0 [20.8–25.3]	47.3 [45.0–49.8]
Month ban was announced (Jan 24)	3.8 [3.1–4.7]	21.3 [19.2–23.6]	34.4 [31.7–37.4]	52.1 [43.2–62.9]	30.3 [27.0–34.0]	54.8 [51.2–58.5]
1 year post‐announcement (Jan 25)	4.0 [3.1–5.2]	21.0 [18.6–23.8]	31.8 [28.5–35.5]	39.1 [30.9–49.5]	20.8 [17.4–25.0]	36.8 [32.6–41.5]
Planned implementation of ban (June 25)	–	–	–	34.7 [25.6–47.0]	17.8 [13.6–23.5]	31.2 [26.0–37.4]

Abbreviations: Jan, January; RR, risk ratio.

Results shown are derived from segmented regression analyses (using generalised additive models) of data collected from January 2022 to January 2025. Models are adjusted for seasonality.

^a^ Among all respondents (unweighted *n*: 51715 never smokers, 23 269 former smokers, 12 614 current smokers).

^b^ Among respondents who reported current vaping in waves that assessed the main device type used (unweighted *n*: 1035 never smokers, 3556 former smokers, 3319 current smokers).

## DISCUSSION

Since the government announced its intention to ban disposable vapes and to introduce other potential vaping restrictions in January 2024, there have been two important changes in vaping trends in Great Britain. First, people have increasingly opted to mainly use reusable rather than disposable vapes. Based on current trends, we estimate that the proportions of adult and young adult vapers mainly using disposable vapes will have approximately halved between the announcement of the ban in January 2024 and its implementation in June 2025. Second, recent increases in vaping prevalence have stalled—including among young adults. These changes were similar across current, former and never regular smokers.

Our findings provide some reassurance that vaping rates are no longer continuing to rise rapidly. Therefore, the stricter policy options under the Tobacco and Vapes Bill are not necessary to halt the rapid rise in vaping, but some restrictions may be needed to bring the high rates of vaping that persist down further. While vapers shifting away from disposable products is likely to have a positive impact on reducing environmental harms [10], it does mean that a ban on disposable vapes is unlikely to have a large impact on reducing vaping prevalence. Other policy measures are likely to be needed to make vaping less appealing to young people. It will be important to weigh the need for tighter regulation of vaping to reduce appeal to young people against any unintended consequences for smokers (who may benefit from using e‐cigarettes to stop smoking [28]) and for ex‐smokers who vape (for whom vaping provides a less harmful alternative to smoking [29]).

As our analysis demonstrates, Great Britain has detailed and regular monitoring of vaping and smoking outcomes that can provide real‐time surveillance and allow policy decisions to be informed by the latest available evidence. However, several limitations should be considered. While the total sample size was large, the number of vapers per wave was relatively small. The repeat cross‐sectional design allows tracking of changes in the prevalence of the main device type used by vapers, but not the extent to which individuals are switching from disposable to reusable vapes over time (which would require longitudinal data). The survey only captures the main device type used by vapers. In reality, numbers using disposable vapes will be higher given that some people will use both disposable and reusable vapes. It is also possible that there was some under‐reporting of disposable use following the announcement of the ban. A decline in use is consistent with the decline in Medicines and Healthcare products Regulatory Agency notifications for disposable vapes [23], but triangulation with sales data would be useful. Finally, our data cannot determine cause and effect, and it is unclear whether the changes in vaping we observed were the result of the announcement of the proposed policy changes or other factors. It is possible that the announcements, and all the widespread accompanying and subsequent media coverage on vaping, signalled to people that these products were less appealing than they previously believed and deterred people from starting to vape who otherwise may have taken it up. There is precedent for changes in behaviour in anticipation of new policies ahead of their implementation [22]. In terms of the decline in the use of disposables among people who vape, the market may have responded to the upcoming ban legislation by producing a range of similar ‘reusable disposable’ products that would not be affected by the ban [23], and people shifted to those products. Despite the limitations of this analysis, our data provide useful insights into changes in vaping prevalence and product choice in a rapidly evolving policy landscape.

## AUTHOR CONTRIBUTIONS

**Sarah E. Jackson:** Conceptualization (lead); formal analysis (lead); investigation (equal); methodology (equal); visualization (lead); writing—original draft (lead); writing—review and editing (equal). **Lion Shahab:** Funding acquisition (equal); investigation (equal); methodology (equal); writing—review and editing (equal). **Harry Tattan‐Birch:** Investigation (equal); methodology (equal); writing—review and editing (equal). **Vera Buss:** Data curation (lead); investigation (equal); methodology (equal); writing—review and editing (equal). **Jamie Brown:** Data curation (supporting); funding acquisition (equal); investigation (equal); methodology (equal); supervision (lead); writing—review and editing (equal).

## DECLARATION OF INTERESTS

J.B. has received unrestricted research funding from Pfizer and J&J, who manufacture smoking cessation medications. L.S. has received honoraria for talks, unrestricted research grants and travel expenses to attend meetings and workshops from manufactures of smoking cessation medications (Pfizer; J&J) and has acted as paid reviewer for grant awarding bodies and as a paid consultant for health care companies. All authors declare no financial links with tobacco companies, e‐cigarette manufacturers, or their representatives.

## ETHICS APPROVAL

Ethical approval for the STS was granted originally by the UCL Ethics Committee (ID 0498/001). The data are not collected by UCL and are anonymised when received by UCL.

## Data Availability

Data are available on Open Science Framework (https://osf.io/fsudk/).

## References

[1] Tattan‐Birch H , Brown J , Shahab L , Beard E , Jackson SE . Trends in vaping and smoking following the rise of disposable e‐cigarettes: a repeat cross‐sectional study in England between 2016 and 2023. Lancet Reg Health ‐ Eur. 2024;42:100924. 10.1016/j.lanepe.2024.100924 39070753 PMC11281926

[2] Action on Smoking and Health . Use of vapes (e‐cigarettes) among adults in Great Britain. 2024 https://ash.org.uk/resources/view/use-of-e-cigarettes-among-adults-in-great-britain (accessed 11 Nov 2024).

[3] Action on Smoking and Health . Use of vapes (e‐cigarettes) among young people in Great Britain. 2024 https://ash.org.uk/resources/view/use-of-e-cigarettes-among-young-people-in-great-britain (accessed 11 Nov2024).

[4] Jackson SE , Tattan‐Birch H , Shahab L , Brown J . Trends in long term vaping among adults in England, 2013–23: population based study. BMJ. 2024;386:e079016. 39019543 10.1136/bmj-2023-079016 PMC11253215

[5] Tattan‐Birch H , Jackson SE , Kock L , Dockrell M , Brown J . Rapid growth in disposable e‐cigarette vaping among young adults in Great Britain from 2021 to 2022: a repeat cross‐sectional survey. Addiction. 2023;118(2):382–386. 10.1111/add.16044 36065820 PMC10086805

[6] Jackson SE , Tattan‐Birch H , Shahab L , Oldham M , Kale D , Brose L , et al. Who would be affected by a ban on disposable vapes? A population study in Great Britain. Public Health. 2024;227:291–298. 10.1016/j.puhe.2023.12.024 38267284

[7] Action on Smoking and Health . Policy options to tackle the issue of disposable (singleuse) vapes. 2023.

[8] Smith MJ , MacKintosh AM , Ford A , Hilton S . Youth's engagement and perceptions of disposable e‐cigarettes: a UK focus group study. BMJ Open. 2023;13(3):e068466. 10.1136/bmjopen-2022-068466 PMC10040067 36948552

[9] Department of Health, Neil O'Brien MP . Crackdown on illegal sale of vapes. 2023 https://www.gov.uk/government/news/crackdown-on-illegal-sale-of-vapes (accessed 14 Sep2023).

[10] Smith L , Sutherland N . Environmental impact of disposable vapes. 2023.https://commonslibrary.parliament.uk/research-briefings/cdp-2022-0216/ (accessed 15 Dec2023).

[11] Local Government Association . Councils call for ban of disposable vapes. 2023. https://www.local.gov.uk/about/news/councils-call-ban-disposable-vapes (accessed 14 Sep2023).

[12] Ferguson D . Ministers set to ban single‐use vapes in UK over child addiction fears The Guardian; 2023 https://www.theguardian.com/society/2023/sep/11/ban-on-single-use-vapes-in-uk-could-be-imminent (accessed 14 Sep2023).

[13] Mahase E . Paediatricians call for ban on disposable e‐cigarettes as child vaping rises. BMJ. 2023;381:p1266. 10.1136/bmj.p1266 37279986

[14] Department of Health and Social Care . Stopping the start: our new plan to create a smokefree generation. 2023 https://www.gov.uk/government/publications/stopping-the-start-our-new-plan-to-create-a-smokefree-generation (accessed 5 Oct2023).

[15] Department of Health and Social Care, Prime Minister's Office, 10 Downing Street , Department for Environment, Food & Rural Affairs, HM Revenue & Customs , The Rt Hon Andrea Leadsom MP . Disposable vapes banned to protect children's health GOV.UK; 2024 https://www.gov.uk/government/news/disposable-vapes-banned-to-protect-childrens-health (accessed 26 Feb2024).

[16] Disposable vapes to be banned for children's health, government says. BBC News. 2024.https://www.bbc.com/news/uk-68123202 (accessed 16 Dec2024).

[17] Hansard . Environmental protection volume 756: debated on Wednesday 13 November 2024 UK Parliam; 2024 https://hansard.parliament.uk/commons/2024-11-13/debates/CABFF1BC-018C-4D72-AE6C-D557B598704D/EnvironmentalProtection (accessed 16 Dec2024).

[18] Department for Environment, Food & Rural Affairs , Department of Health and Social Care , Mary Creagh CBE MP , Andrew Gwynne MP . Government crackdown on single‐use vapes GOV.UK; 2024 https://www.gov.uk/government/news/government-crackdown-on-single-use-vapes (accessed 11 Nov2024).

[19] Scotland disposable vape ban delayed to bring it in line with UK. BBC News 2024.https://www.bbc.com/news/articles/cp8x1gyg0lro (accessed 16 Dec2024).

[20] E‐cigarettes: single‐use vapes to be banned in Wales. BBC News. 2024.https://www.bbc.com/news/articles/c7043578863o (accessed 16 Dec2024).

[21] Department of Health and Social Care . Tobacco and Vapes Bill v2. 2024 https://bills.parliament.uk/bills/3879 (accessed 11 Nov2024).

[22] Opazo Breton M , Britton J , Brown J , Beard E , Bogdanovica I . Was the implementation of standardised tobacco packaging legislation in England associated with changes in smoking prevalence? A segmented regression analysis between 2006 and 2019. Tob Control. 2021; 32:195‐204. PMID: tobaccocontrol‐2021–056694. 10.1136/tobaccocontrol-2021-056694 34326193

[23] Copland C . UK regulations & enforcement. In: The E‐cigarette summit UK, 2024 London, UK; 2024 https://www.e-cigarette-summit.co.uk/programme-2024/ (accessed 19 Dec2024).

[24] Fidler JA , Shahab L , West O , Jarvis MJ , McEwen A , Stapleton JA , et al. ‘The smoking toolkit study’: a national study of smoking and smoking cessation in England. BMC Public Health. 2011;11(1):479. 10.1186/1471-2458-11-479 21682915 PMC3145589

[25] Kock L , Shahab L , Moore G , Beard E , Bauld L , Reid G , et al. Protocol for expansion of an existing national monthly survey of smoking behaviour and alcohol use in England to Scotland and Wales: the smoking and alcohol toolkit study. Wellcome Open Res. 2021;6:67. 34458587 10.12688/wellcomeopenres.16700.1 PMC8370132

[26] Jackson SE , Beard E , Kujawski B , Sunyer E , Michie S , Shahab L , et al. Comparison of trends in self‐reported cigarette consumption and sales in England, 2011 to 2018. JAMA Netw Open. 2019;2(8):e1910161. 10.1001/jamanetworkopen.2019.10161 31461148 PMC6716287

[27] Deville J‐C , Särndal C‐E , Sautory O . Generalized raking procedures in survey sampling. J am Stat Assoc. 1993;88(423):1013–1020. 10.1080/01621459.1993.10476369

[28] Lindson N , Butler AR , McRobbie H , Bullen C , Hajek P , Begh R , et al. Electronic cigarettes for smoking cessation. Cochrane Database Syst Rev. 2024;2024(1):CD010216. 10.1002/14651858.CD010216.pub8 PMC8094228 33052602

[29] McNeill A , Simonavicius E , Brose LS , Taylor E , East K , Zuikova E . Nicotine vaping in England: an evidence update including health risks and perceptionsSeptember 2022. A report commissioned by the Office for Health Improvement and Disparities London: Office for Health Improvement and Disparities; 2022 https://www.gov.uk/government/publications/nicotine-vaping-in-england-2022-evidence-update (accessed 3 Oct2022).

